# One Health drivers of antibacterial resistance: Quantifying the relative impacts of human, animal and environmental use and transmission

**DOI:** 10.1016/j.onehlt.2021.100220

**Published:** 2021-01-26

**Authors:** Ross D. Booton, Aronrag Meeyai, Nour Alhusein, Henry Buller, Edward Feil, Helen Lambert, Skorn Mongkolsuk, Emma Pitchforth, Kristen K. Reyher, Walasinee Sakcamduang, Jutamaad Satayavivad, Andrew C. Singer, Luechai Sringernyuang, Visanu Thamlikitkul, Lucy Vass, OH-DART Study Group, Matthew B. Avison, Nour Alhusein, Ross D. Booton, Henry Buller, Boonrat Chantong, Nisanart Charoenlap, Natacha Couto, Punyawee Dulyayangkul, Edward Feil, Marjorie J. Gibbon, Virginia C. Gould, Helen Lambert, Aronrag Meeyai, Skorn Mongkolsuk, Varapon Montrivade, Emma Pitchforth, Kornrawan Phoonsawad, Nuchanart Rangkadilok, Parntep Ratanakorn, Kristen K. Reyher, Walasinee Sakcamduang, Jutamaad Satayavivad, Andrew C. Singer, Kwanrawee Sirikanchana, Luechai Sringernyuang, Tawit Suriyo, Sarin Suwanpakdee, Visanu Thamlikitkul, Katherine M.E. Turner, Lucy Vass, Kantima Wichuwaranan, Anuwat Wiratsudakul, Matthew B. Avison, Katherine M.E. Turner

**Affiliations:** a University of Bristol, UK; b University of Exeter, UK; c Mahidol University, Thailand; d Chulabhorn Research Institute, Thailand; e University of Bath, UK; f London School of Hygiene and Tropical Medicine, UK; g Chulabhorn Royal Academy, Thailand; h Centre for Ecology & Hydrology, UK; a Bristol Veterinary School, University of Bristol, Bristol, UK; b Department of Epidemiology, Mahidol University, Bangkok, Thailand; c Department of Global Health and Development, London School of Hygiene and Tropical Medicine, UK; d Bristol Medical School, University of Bristol, Bristol, UK; e College of Life and Environmental Sciences, University of Exeter, Exeter, UK; f Department of Biology & Biochemistry, University of Bath, Bath, UK; g Laboratory of Biotechnology, Chulabhorn Research Institute, Bangkok, Thailand; h College of Medicine and Health, University of Exeter, Exeter, UK; i Faculty of Veterinary Science, Mahidol University, Nakhon Pathom, Thailand; j Laboratory of Pharmacology, Chulabhorn Research Institute, Bangkok, Thailand; k UK Centre for Ecology & Hydrology, Wallingford, UK; l Faculty of Social Sciences and Humanities, Mahidol University, Bangkok, Thailand; m Faculty of Medicine Siriraj Hospital, Mahidol University, Bangkok, Thailand; n School of Cellular & Molecular Medicine, University of Bristol, Bristol, UK

**Keywords:** Antibacterial resistance, Mathematical model, One health, Transmission, Antibacterial usage, Thailand

## Abstract

**Objectives:**

Antibacterial resistance (ABR) is a major global health security threat, with a disproportionate burden on lower-and middle-income countries (LMICs). It is not understood how ‘One Health’, where human health is co-dependent on animal health and the environment, might impact the burden of ABR in LMICs. Thailand's 2017 “National Strategic Plan on Antimicrobial Resistance” (NSP-AMR) aims to reduce AMR morbidity by 50% through 20% reductions in human and 30% in animal antibacterial use (ABU). There is a need to understand the implications of such a plan within a One Health perspective.

**Methods:**

A model of ABU, gut colonisation with extended-spectrum beta-lactamase (ESBL)-producing bacteria and transmission was calibrated using estimates of the prevalence of ESBL-producing bacteria in Thailand. This model was used to project the reduction in human ABR over 20 years (2020–2040) for each One Health driver, including individual transmission rates between humans, animals and the environment, and to estimate the long-term impact of the NSP-AMR intervention.

**Results:**

The model predicts that human ABU was the most important factor in reducing the colonisation of humans with resistant bacteria (maximum 65.7–99.7% reduction). The NSP-AMR is projected to reduce human colonisation by 6.0–18.8%, with more ambitious targets (30% reductions in human ABU) increasing this to 8.5–24.9%.

**Conclusions:**

Our model provides a simple framework to explain the mechanisms underpinning ABR, suggesting that future interventions targeting the simultaneous reduction of transmission and ABU would help to control ABR more effectively in Thailand.

## Highlights

• We present a novel mathematical model of human, animal and environment ABR transmission and ABU.
• Human ABU was identified as the main driver of human ABR.
• Resistance within humans was primarily driven by human activity rather than animal or environmental usage or transmission.
• One Health interventions which consider human, animal and environmental transmission and usage can yield the highest impact.

## 1 Introduction

Antimicrobials have played an important role in the treatment and prevention of infectious diseases, have enabled food production to intensify and have greatly improved the lives of many millions of people. However, the emergence and spread of antimicrobial resistance (AMR) threatens to undermine this progress, with drug-resistant pathogens projected to cause ten million annual deaths by 2050 [1]. Hence AMR is regarded as a major global health security issue [2], with many member states of the World Health Organization adopting national action plans (with different stages of financing and implementation) in order to tackle the growing threat of AMR [3].

AMR, particularly Antibacterial Resistance (ABR), occurs at the interface of a multifaceted One Health system; human health is thought to not only depend on the human population's health-related behaviour, but also on industrial, farming and veterinary practices as well as environmental conditions [4]. In the context of ABR, these diverse drivers can be separated into two components: “selection”, predominantly by antibacterial use (ABU) and “transmission” of resistant organisms between each connected compartment on a human-animal-environment axis. In terms of selection, the majority of global ABU is within animals raised for food (73%, [5,6]) and it is generally accepted that ABU in animals drives ABR [7], although the magnitudes of these effects are poorly characterised and are likely to be antibacterial, resistance mechanism and organism specific. However, ABU within human populations (of which up to 50% has been suggested to be unnecessary [8]) is also a fundamental driver of ABR [9]. In terms of transmission, sharing of resistant bacteria between humans, animals and the environment can occur via human-human contact (open community, contact with patients, household transmission, contact in workplaces, travellers), human-animal contact (occupational contact with farm animals, food consumption or preparation), animal-animal contact (relating to farming practices, or movement of wild or domesticated animals), and human-environment or animal-environment interfaces (sewage and manure, habitat, drinking water, bathing, leisure activities, food sources and soil) [10]. Crucially, we do not know the full extent to which the listed factors of selection and transmission lead to the currently observed growing prevalence of ABR and increasing incidence of drug-resistant infections.

Selection and transmission of ABR are not entirely independent. ABU drives selection of pre-existing resistant bacteria through population-level mechanisms [11], while simultaneously selecting for successful transmission of resistance into the bacterial population (either via the transmission of mobile genetic elements between microorganisms or through direct transmission of the microorganisms themselves) [7]. Therefore, while ABU in humans and animals, contamination of the environment from those sources or ABU within non-animal agriculture are thought to generally increase the prevalence of ABR, the relationship between ABU and resistance is highly complex and dependent on pre-existing bacterial population structures [7]. To account for this, previous studies have suggested that no single *‘silver bullet’* solution exists. Rather, that preventing and reducing the burden of ABR within a One Health system should take a multifactorial, coordinated approach focussing on the specifics of ABU, and the types and prevalence of ABR in each system, while considering the potential interactions within and between compartments [7]. It should also take a multi-sector, transdisciplinary, collaborative approach. One Health is a relatively recent global policy framing of ABR [12] and to date, while animal health has been increasingly included in national policies and action plans, the environment has been given less emphasis [13,14]. One Health approaches are promoted widely in the field, yet the relative contributions of different drivers or the impacts of different interventions are not known, and remain unquantified [10]. Quantification could aid the prioritisation of interventions and refine policy approaches in the inherently complex field of ABR.

While ABR is a global issue [15], there is a disproportionate burden of infectious diseases in lower- and middle-income countries (LMICs) [16]. While these countries experience higher rates of infection (up to three times greater than high income countries [17]), emerging evidence suggests the burden of ABR is greater in LMICs [18] while simultaneously there is limited access to essential antibacterials [8]. Perhaps counterintuitively, research focussed on modelling ABR within lower- and middle-income Southeast Asian countries is vastly underrepresented when compared to European or African studies (eight published models in South-East Asia, 35 in Africa and 42 in Europe [19]), especially when considering that the prevalence of extended-spectrum beta-lactamase (ESBL)-producing *Escherichia coli* (i.e. a key resistance mechanism and pathogen relevant to human and animal health and a sentinel for One Health ABR) in Southeast Asia is high (22% in Southeast Asia compared to 4% in Europe [18]).

Here we focus on the specific national setting of Thailand, an upper-middle income country in Southeast Asia with a high burden of ABR relative to other countries [20] which affects both human health (88,000 infections, 38,000 deaths attributed to ABR per year in 2010) and the economy (direct costs of $70–170 million to treat ABR, indirect costs at least $1100 million for morbidity in 2010) [21]. The prevalence of faecal colonisation with ESBL-producing *E. coli* among healthy humans in selected communities in Thailand has grown from 0% in 2004 [22] to 69% in 2010 [23] and 74% in 2012 [24]. In response to the threat posed by rising ABR, the Thai cabinet implemented their first five-year policy in 2016, the “National Strategic Plan on Antimicrobial Resistance in Thailand” (NSP-AMR) running until 2021 [25]. The NSP-AMR reflects the strategic objectives of the WHO Global Action Plan [3] and aims to reduce AMR morbidity in Thailand by 50% through 20% reductions in AMU in humans, 30% reductions in AMU in animals and 20% increases in public knowledge on AMR (including awareness of AMU) by 2021 [25].

We aim to quantify the relative contributions to the human ABR burden (% colonisation with resistant bacteria) of human, animal and environmental factors (including ABU and transmission of ABR bacteria) within a One Health system in Thailand. We propose a simple mathematical framework for the spread of ABR between these compartments from which we will explore and assess national interventions aimed at reducing ABR. Our objective is to assess and compare a wide variety of One Health drivers and provide insights into the multifaceted problem of ABR. This simple model is intended to stimulate further discussion on how best to reduce the burden of ABR in human populations and to provide the much needed first step in providing a workable One Health modelling framework.

## 2 Materials and methods

We built a compartmental model of ordinary differential equations (ODEs) to describe the relationship between resistant bacteria in three compartments: humans, animals and the environment. We considered the fraction of all humans colonised with resistant bacteria (H), the fraction of animals with resistant bacteria (A) and the fraction of environmental samples with resistant bacteria (E) based on the framework of a previously published model of animal-human transmission [26]. Here, we assume that the bacteria are ESBL-producing *E. coli*, and human/animal colonisation is assumed to be of the gut and via the faecal-oral route. Given our use of a sentinel pre-evolved resistance mechanism, de novo selection of resistance within a compartment is not considered significant.

We assumed that resistance develops from two sources; antibacterial use (Box 1A, which selects resistant bacteria already present in the host) and transmission from other compartments (Box 1B, which is also dependent on antibacterial use in those compartments).

First, resistance develops in humans from exposure to antibacterials, proportional to their usage in medicine Λ_H_, the rate at which humans are colonised with resistant bacteria, *γ*, and the fraction of humans not already colonised (1 − *H*). In a similar fashion, resistance develops in animals proportional to antibacterial usage in animal, veterinary and farm practices Λ_A_, the rate at which animals are colonised with resistant bacteria, *γ*, and the proportion of animals not already colonised (1 − *A*) (Box 1A).

Λ_E_ represents the presence of antibacterial in the environment (derived from factories, pollution, wastewater etc), while (1 − *E*) represents the proportion of environmental samples negative for ABR bacteria. We assumed that the prevalence (percent of humans, animals or environmental samples that have resistant bacteria) of ABR bacteria declined at rates *μ*_*H*_, *μ*_*A*_ and *μ*_*E*_ within humans, animals and the environment respectively.

**Box 1:**
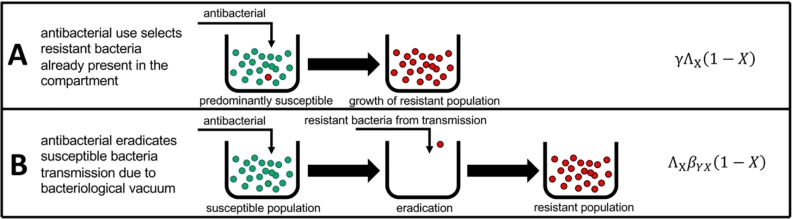
Two mechanisms of resistance driven by antibacterial exposure. First, antibacterial use selects resistant bacteria already present in the compartment (A), and second that antibacterial use can remove susceptible populations making transmission more likely (B). In (B), it is more likely for colonisation with resistant bacteria to occur when the native flora have been removed (via a ‘bacteriological vacuum’ - an absence of bacteria).

Second, we assumed that humans negative for ABR bacteria are exposed to and acquire resistant bacteria from other humans at rate *β*_*HH*_ (representing transmission in the open community and from patients, farm workers and other high-risk groups). Acquisition in this context means the combined effects of stable colonisation with a resistant bacterium following transfer, and infiltration of mobile resistance genes (e.g. via plasmid) into the existing bacterial flora following transient colonisation with a transferred resistant bacterium. Similarly, animal-to-animal acquisition of resistance occurs at rate *β*_*AA*_, and animal-to-human transfer of resistance occurs at rate *β*_*AH*_ (representing contact with animals and food consumption). Human-to-animal transmission of resistance occurs at rate *β*_*HA*_, however this rate is smaller than the animal-to-human rate *β*_*AH*_ (as livestock to human jumps occur more frequently over evolutionary history than vice versa [10]). Similarly, the rates of acquisition of resistance occur at *β*_*EH*_ for environment-to-human (drinking water, non-animal food sources, swimming and bathing in freshwater), *β*_*EA*_ for environment-to-animal (habitat, food sources, drinking water), *β*_*HE*_ for human-to-environment (transfer through sewage) and *β*_*AE*_ for animal-to-environment (manure, or composting of dead animals). We assumed that transmission from the environment to animals was greater than that of the environment to humans (*β*_*EA*_ > *β*_*EH*_), as a previous study showed that there were higher proportions of shared bacterial genera in wastewater and animals, as opposed to shared bacterial genera in wastewater and humans [27]. We also assumed that the transmission within populations of humans and animals is greater than the transmission between these populations and the transmission from the environment to these populations (*β*_*HH*_ > *β*_*AH*_, *β*_*EH*_ and *β*_*AA*_ > *β*_*HA*_, *β*_*EA*_). These assumptions are summarised in Table S2.

**Fig. 1:**
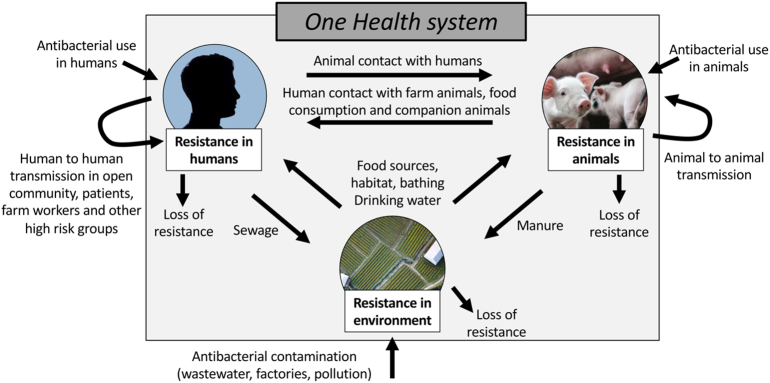
Model schematic for human-animal-environment transmission of ABR in the One Health setting.

We assumed that all rates of transmission are proportional to exposure with antibiotics, due to treatment eradicating susceptible bacteria in the host thereby enabling colonisation by incoming resistant bacteria (Box 1B, [11]). Therefore, the dynamics for the fraction of humans, animals and the environment with resistant bacteria is represented by the following ordinary differential equation model (Fig. 1):

$$\frac{\mathrm{dH}\;}{\mathrm{dt}}={\gamma \Lambda }_{H}\left(1-H\right)+\Lambda_{H}\beta_{\mathrm{HH}}H\left(1-H\right)+\Lambda_{H}\beta_{\mathrm{AH}}A\left(1-H\right)+\Lambda_{H}\beta_{\mathrm{EH}}E\left(1-H\right)-\mu_{H}H$$

$$\frac{\mathrm{dA}\;}{\mathrm{dt}}={\gamma \Lambda }_{A}\left(1-A\right)+\Lambda_{A}\beta_{\mathrm{AA}}A\left(1-A\right)+\Lambda_{A}\beta_{\mathrm{HA}}H\left(1-A\right)+\Lambda_{A}\beta_{\mathrm{EA}}E\left(1-A\right)-\mu_{A}A\;$$

$$\frac{\mathrm{dE}\;}{\mathrm{dt}}=\Lambda_{E}\beta_{\mathrm{EE}}E\left(1-E\right)+\Lambda_{E}\beta_{\mathrm{HE}}H\left(1-E\right)+\Lambda_{E}\beta_{\mathrm{AE}}A\left(1-E\right)-\mu_{E}E$$

For example, the rate of change over time for the fraction of humans with resistant bacteria $\;\frac{\mathrm{dH}\;}{\mathrm{dt}}\;$= the rate at which susceptible humans become colonised with resistant bacteria γΛ_H_(1 − *H*) + the transmission from humans with ABR to susceptible humans Λ_*H*_*β*_*HH*_*H*(1 − *H*) + the transmission from animals with ABR to susceptible humans Λ_*H*_*β*_*AH*_*A*(1 − *H*) + the transmission of resistant bacteria in the environment to susceptible humans Λ_*H*_*β*_*EH*_*E*(1 − *H*) – the loss of resistance *μ*_*H*_*H* at each time point.

We assumed that Λ_A_ > Λ_*H*_ as the majority of global ABU is within animals raised for food (73%, [5,6]). We further assumed that ABU is greater in humans when compared to the amounts present in the environment Λ_H_ > Λ_*E*_, and that the rates of loss of resistance are equal in all three settings (*μ*_*H*_ = *μ*_*A*_ = *μ*_*E*_) in the absence of data on decay of resistance within different compartments.

The model was coded and numerically simulated using R v.1.2.5019. ODEs were solved using functions *deSolve* and *ode* in R.

We identified estimates (Table 1) for the prevalence of gut colonisation with ESBL-producing *E. coli* in Thailand for healthy humans across an eight-year timeframe (2004–2012) [[22], [23], [24],^28^,^29^], estimates of sample level positivity for resistant bacteria in rectal swabs from animals and fresh food from Thailand in 2012–2013 [24], and environmental estimates of the proportion of bacteria that are resistant in Thailand from canal water sources [24], stagnant water on food animal farms, and liquid from hospital wastewater treatment tanks [30]. These data were used to calculate lower and upper bounds (95% credible intervals) in order to calibrate our model to a national-level Thailand-specific setting.

**Table 1:** Parameters and fitting metrics used in the model (summary of 95% confidence interval uncertainty ranges). HH = human-human, AH = animal-human, AA = animal-animal.

Model Parameters	Parameter value (95% confidence interval)	Source
Antibacterial usage		
Animals Λ_A_	0–100%	Majority of antimicrobials used in animals raised for food (73%, [5,6])
Humans Λ_H_	0–100% of animal use	
Environment Λ_E_	0–100% of human use	
Transmission of ABR		
Humans to humans *β*_*HH*_	0.0–100.0%	Explore full parameter space
Animals to humans *β*_*AH*_	0.0–100.0% of HH	Fraction of *β*_*HH*_
Environment to humans *β*_*EH*_	0.0–100.0% of HH	Fraction of *β*_*HH*_
Animals to animals *β*_*AA*_	0.0–100.0%	Explore full parameter space
Humans to animals *β*_*HA*_	0.0–100% of AH and AA	Assume wide range, but less than *β*_*AH*_ [10] and *β*_*AA*_ transmission
Environment to animals *β*_*EA*_ (habitat/ drinking water)	0.0–100.0% of AA	Fraction of *β*_*AA*_
Humans to environment *β*_*HE*_ (sewage)	0.0–100.0%	Explore full parameter space
Animals to environment *β*_*AE*_ (manure)	0.0–100.0%	Explore full parameter space
Carriage of resistance		
Rate of loss of resistant bacteria after 12 months *μ*_*H*_ = *μ*_*E*_ = *μ*_*A*_	0–100%	Explore full parameter space
Fitting Parameters	Value (95% confidence interval)	Source
Prevalence of colonisation with ESBL-producing organisms		
Humans		
2004	0.0–0.0%	0/120 healthy adults in Thailand [22]
2008	46.7–62.1%	87/160 healthy asymptomatic volunteers in Thailand [28]
2009 (not included in fitting)	35.2–44.3%	177/445 healthy asymptomatic volunteers in Thailand [29]
2010	64.9–73.7%	289/417 door to door sampling among healthy individuals in Thailand [23]
2012	71.4–78.5%	430/574 healthy workers in a food factory and food animal farm in Thailand [24]
Animals	11.1–60.3%	Upper bound from rectal swabs in pigs (241/400), lower bound from fresh food (12/54) in Bangkok and East/North Thailand [24]
Rectal swabs of animals, 2012–2013		
Pig	55.4–65.0%	241/400 in East/North Thailand [24]
Broiler	26.9–48.1%	30/80 in East/North Thailand [24]
Laying Hen	0.0–7.7%	2/61 in East/North Thailand [24]
Fresh pork meat	0.0–25.6%	2/18 in East/North Thailand [24]
Fresh food, 2012–2013		
Chicken	4.9–52.2%	4/14 in Bangkok & central [24]
Pork	28.1–78.6%	8/15 in Bangkok & central [24]
Beef	0.0–0.0%	0/11 in Bangkok & central [24]
Fish	0.0–0.0%	0/14 in Bangkok & central [24]
Environment	0.0–24.7%	Lower bound from water in Bangkok and central province [24] and upper bound from stagnant water on food animal farms [24]
Canal water, 2012–2013	0.0–19.3%	1/15 in Bangkok and central [24]
Stagnant water on food animal farms, 2012–2013	0.0–24.7%	3/25 in Bangkok and central [24]
Effluent fluid after treatment with chlorine prior to draining into a public water source from hospitals	2.4–17.6%	6/60 in public hospitals in Thailand [30]

We assumed the widest possible range for parameters with no prior data and extrapolated based on the hierarchy of transmission (e.g. human-human transmission is greater or equal to animal-human transmission). Therefore, we allowed human-human transmission to take any possible value (from 0 to 100%), while animal-to-human transmission is a fraction of this value (0–100% of human-human). We explored the full range of transmission for animal-animal, animal-environment and human-environment, while environment-human is a fraction of human-human, and human-animal and environment-animal are a fraction of the animal-animal rate (Table 1). This method ensures that we explored every possible scenario while maintaining the structure of the model and the hierarchy of the separate transmission rates.

Similarly, we allowed ABU within animals to vary between the minimum and maximum values (0–100%), while human use is a fraction of this (less than or equal to animal use), and environmental use or contamination is a fraction of human use (Table 1).

Using this available data, we defined ranges for all parameters in the model (Table 1), and sample between these ranges using Latin Hypercube Sampling (a statistical method for generating random parameters from multidimensional data), for a total of 1,000,000 simulations/parameter sets. For each simulation we output the prevalence in each year for each compartment, accepting this parameter set if the output falls within the ranges of prevalence for humans in 2004, 2008, 2010 and 2012, animals in 2012 and the environment in 2012.

We also explore an additional analysis with a simple extension of the model in the supplementary information. This was performed to investigate the model's sensitivity to the assumptions relating to transmission (and in particular when there is an absence of antibiotic).

## 3 Results

**Fig. 2:**
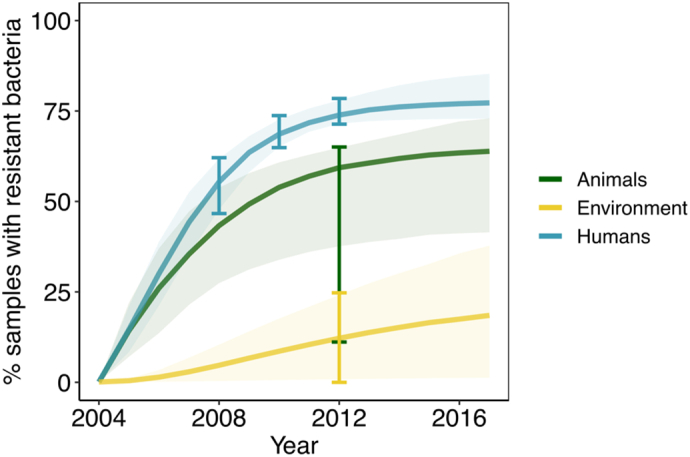
Comparison of the model projections for animals, humans and the environment for the best 431 model fits (from 1 million samples). Error bars indicate data from which the model was calibrated (3/4 for humans, aside from 2009) and shaded areas indicate the 95% confidence interval among all fits. Model parameters and their uncertainty ranges can be found in Table 1.

From 1,000,000 simulated parameter sets, 431 agreed well with the lower and upper bounds (Table 1) for the percentage of humans, animals and environmental samples colonised with resistant bacteria in Thailand (Fig. 2). The fitting bounds for the percentage of humans with resistant bacteria was underestimated in 2009, perhaps due to discrepancy in sampling methodology or a different sampling cohort [29]. The full description of the prior and posterior parameter ranges can be found in the supplementary information (Fig. S1). Note that certain priors are defined to be the absolute maximum and minimum possible (i.e., 0–100%) in the absence of data for these parameters.

### 3.1 The national strategic plan on AMR in Thailand

Table 2 shows the potential predicted impact of reduction in colonisation with resistant bacteria in humans via interventions starting in 2020 and running for 20 years until 2040 (compared to the current standard of care). Over 20 years (2020–2040), the NSP-AMR in Thailand (which aims to reduce antimicrobial use in humans by 20%, animals by 30% and increase public knowledge of AMR equating to an increase in all sanitary practices e.g. handwashing, safe drinking water, sewage disposal, waste water treatment plants by 20%) was estimated to reduce the number of humans colonised with resistant bacteria by 12.2% (95% credible interval: 6.0–18.8%, Table 2) from 2020 values by 2040. The NSP-AMR supplemented with an additional reduction in human-based ABU (to 30%) would reduce the burden of resistance (% colonised with resistant bacteria) within humans to a greater extent: to 16.7% (8.5–24.9%, Table 2) of 2020 values. The NSP-AMR supplemented by an additional increase in sanitary practices (to 30%, we predict an impact similar to reducing ABU by 30%: a reduction of 15.1% (7.4–24.6%, Table 2) from 2020 values.

**Table 2:** Reduction in colonisation with resistant bacteria in humans between 2020 and 2040 for different potential interventions. The National Strategic Plan on Antimicrobial Resistance (NSP-AMR; 2017–2021) in Thailand aims to reduce antibiotic use (ABU) in humans by 20%, animals by 30% and increase sanitary knowledge by 20% (in 2021). We investigate other hypothetical interventions aiming to reduce ABU in humans and animals and other transmission related interventions.

*Intervention or scenario*	*Median reduction in colonisation with resistant bacteria in humans (95% credible interval) 2020–2040*
NSP-AMR, Thailand	12.2 (6.0–18.8)
NSP-AMR with 30% reduction in human ABU	16.7 (8.5–24.9)
NSP-AMR with 30% increase in sanitary practices	15.1 (7.4–24.6)
50% reduction in all transmission only	13.4 (5.7–25.6)
50% reduction in water related transmission only	0.1 (0.0–1.2)
20% reduction in human ABU with 50% reduction in human-human transmission	13.9 (7.9–22.3)
20% reduction in human ABU with 95% reduction in transmission from sewage/manure	6.3 (3.5–8.6)

Without any ABU reduction, reducing all transmission rates in our model to 50% of their original value was predicted to reduce the number of humans carrying resistant bacteria to 13.4% (5.7–25.6%, Table 2) by 2040. However, the impact of reducing only the transmission rates relating to water and the environment (human and animal transmission to and from the environment) on colonisation in humans was relatively low (0.1% reduction, 0.0–1.2%, Table 2). A 20% reduction in human ABU together with a 50% reduction in human-human transmission were projected to reduce the human burden of resistance by 13.9% (7.9–22.3%, Table 2), while a 20% reduction in human ABU with a 95% reduction in sewage or manure transmission was projected to reduce the burden of human resistance by 6.3% over 20 years (3.5–8.6%, Table 2).

Fig. S2 shows the potential impact of simultaneously reducing ABU in humans and animals (in intervals of 10%; 0, 10, 20, 30, 40 and 50%) and increasing sanitary practices (transmission remains at current levels, reducing transmission by 25%, 50% and 75%). In the absence of any sanitary interventions aiming to reduce transmission, achieving the NSP-AMR targets for ABU reduction would result in an impact of 7.4% reductions in human colonisation of resistant bacteria by 2040. Increasing sanitary practices to 25% would result in a 1.9-fold reduction in resistant bacteria colonisation in humans (to 14.2%), while increasing sanitary practices by 50% and 75% would result in larger 3.1-fold (to 23.1%) and 6.5-fold (to 47.8%) reductions in the presence of the current Thailand NSP-AMR targets.

### 3.2 Which factors contribute the most to human ABR?

The maximum potential reduction in colonisation with resistant bacteria for humans from single parameters in the model are summarised in Table 3 (more detail available in Table S1). While removing human ABU (minimum 0% ABU in humans) has the highest potential impact (65.7–99.7% reduction in colonisation of resistant bacteria over 20 years), reducing human-to-human transmission (8.2–36.3%), animal ABU (1.0–16.8%) and animal-to-human transmission (1.1–17.1%) all have considerable potential impact. Environmental contamination and environment-to-human transmission were both predicted to have a smaller impact (<1.8%).

**Table 3:** The maximum reduction in resistant bacteria within human populations when accounting for the effects of usage and transmission parameters. The maximum reduction can be achieved by totally stopping human antibacterial use and human-human transmission, but animal and environmental factors can still contribute significantly to reducing the burden of ABR within humans.

*Parameter*	*Median reduction in colonisation with resistant bacteria in humans (95% credible interval) 2020–2040*
Human ABU	95.4 (65.7–99.7)
Human-to-human transmission	17.1 (8.2–36.3)
Animal ABU	7.1 (1.0–16.8)
Animal-to-human transmission	7.9 (1.1–17.1)
Environment contamination by AB	0.1 (0.0–1.8)
Environment-to-human transmission	0.1 (0.0–1.4)

Table S1 shows the maximum potential reduction in colonisation with resistant bacteria in humans for 30 scenarios, by their relative contributions to human ABR. The top four ranked scenarios were all related to human ABU (95% reduction in colonisation over 20 years in humans). The next highest factors were the rate of loss of resistant bacteria in humans (66.7%), followed by no transmission events (38.5%), simultaneous human-human/human-environment/human-animal transmission (17.9%) and human-human transmission alone (17.1%).

The relative attributable impact on the burden of colonisation of resistant bacteria within human populations of human ABU compared to use in animals was determined to be 13:1 (13 times more impact could be achieved through reducing ABU in humans rather than animals, Table S1). This ratio decreased to 12:1 when accounting for all animal-based transmission. When comparing the relative impact of the human AMR burden to human-human transmission, the ratio was 6:1 (six times more impact of reducing human ABU compared to reducing human-human transmission) and comparing to all transmission routes in the model resulted in a ratio of 2:1 (two times more impact of reducing human ABU compared to reducing all transmission).

### 3.3 Which factors contribute the most to animal and environmental ABR?

The top ranked scenarios for both animals and the environment were all related to ABU in animals and antibacterial contamination of the environment, respectively (Table S1). Interestingly, removing all human usage (human ABU = 0) would result in a 3.1% reduction in animals colonised with resistant bacteria, but a much greater 32.9% of the resistant contamination of bacteria in the environment. Removing animal ABU (animal ABU = 0) resulted in a 95.4% reduction in animals and 29.1% reduction in the environment, while removing environmental antibacterial contamination resulted in 0.8% and 96.0% reductions in colonisation in animals and the environment, respectively (Table S1).

## 4 Discussion

Prior to this study, an AMR literature review found that only 2% of published models (*five from a total of 273*) considered human and animal transmission concurrently, and no published model considered a third environmental setting [19]. To our knowledge, therefore, our study is the first to consider the One Health human-animal-environment axes of ABR. This is an especially important factor to consider as human ABU, animal ABU and environmental antibiotic contamination have all been shown to increase the prevalence of ABR [6,10,32,33]. We propose this simple model as a first step in understanding the complex picture [7] of One Health ABR, but our model framework includes assumptions which should be recognised, and thus these results should be interpreted in light of these assumptions.

### 4.1 Limitations of the model

First, prevalence data we used in the fitting of our model came from the six separate studies available in the literature – each with varying methodologies and cohorts. We did, however, explore the reliability of each of these national Thai estimates from 2004 to 2013 with a 95% confidence interval calculated for each reported data point – ensuring that we accounted for differences between these studies. There is a paucity of data on the transmission of bacteria between the different compartments in any One Health system, and no information at all for the situation in Thailand. Instead, we assumed a hierarchy of transmission based on other published studies from other countries. For example, we used a study of shared bacterial genera (those bacteria from similar taxonomic composition) in Beijing, China [27] to inform the assumption that environment-to-animal transmission is greater or equal to that of environment-to-human transmission. We also used a wide range of potential estimates (with 95% confidence intervals) and explored every possibility of all underlying parameters.

Our model primarily considers ABR bacteria transmitted via the faecal-oral route and those carrying mobile resistance genes (i.e. ESBL-producing *E. coli*, which is a very common sentinel for ABR in a One-Health context) [24]. Whilst bacteria transmitted via faecal oral route make up a high proportion of the World Health Organization list of priority ABR pathogens [34], other types of resistant bacteria (e.g. MRSA) do not have an environmental reservoir, and indeed require close physical contact in order to transmit – so this model does not capture those resistance mechanisms of *all* resistant bacteria. Instead we focus on those bacteria which simultaneously affect all aspects of the One Health network [24]. The lack of fit-for-purpose data to inform this model may have skewed our results to favour humans as the most important factor (as this is where the data is most rich), and future data may clarify the complete role of animals and the environment in human ABR. Future models should extend our framework to these aspects and especially consider the setting-specific features of the population and their behaviour. This would result in a better understanding of the One Health drivers of ABR in human populations.

Additionally, our simple framework made assumptions relating to how antibacterials interact with the transmission of resistant bacteria, with the absence of the antibacterial resulting in minimal transmission. This is, of course, an oversimplification of the underlying biological mechanisms of resistance. However, we argue that this is the simplest possible set of assumptions which capture antibacterial usage and transmission of resistance, and their potential interaction. The sensitivity to this assumption was explored in the supplementary information (Table S3), where the addition of a new transmission parameter (transmission independent of antibacterial usage) was shown to lead to small reductions in the overall impact presented in the main text. The inclusion of this additional transmission parameter should be investigated further in future theoretical modelling studies. In addition, we did not consider de novo selection of resistance to be significant throughout our study, however, future work should also investigate relaxing this assumption to include different mechanisms of selection.

### 4.2 Applications of the model

Widespread concern around the contribution of animal antibacterials to human resistance is growing, perhaps due to the majority of use, globally, occurring within this sector [5,6], but the benefits of curtailing their use on human health remain unquantified [10]. Here, the potential absolute impact of limiting the use of antibacterials in animals was predicted to be far less than then limiting human medical use (13 times more impact achieved through reducing ABU in humans when compared to animals), but this ratio was decreased when transmission between animals and humans was accounted for (12 times more impact). This result suggests that while animal ABU has been highlighted as a major driver within human ABR [5], it is far more effective to reduce human ABU in the first instance. However, reducing human ABU may not be feasible in many cases: indeed, reducing animal ABU would also contribute to minimising the development of ABR within animals (67% to 100%, Table S1) with substantial knock-on implications for human health (up to an impact of 17% reductions by 2040). This suggests that animal antibacterials are still an important driver for human resistance (albeit not as important as human use). Reducing animal use of antibiotics should be considered carefully for welfare reasons, even when making minor reductions in use (this model does not suggest we should reduce use by 100%, rather that these results indicate which areas of the One Health system yield maximum potential impact).

Limiting human ABU was six times as effective as reducing human-human transmission and twice as effective as reducing all transmission events. This suggests that interventions targeting reductions in ABR within human populations should also focus on improved hygiene and infection control (particularly for humans) in addition to curtailing ABU.

Overall, therefore, we predict that the most effective method of reducing the burden of resistant bacteria in humans is to combine reductions in ABU while simultaneously reducing transmission events between humans, animals and the environment, reinforcing the need for One Health approaches that consider all three. This finding agrees with the results of other studies: one such study found that animal ABU alone had little impact on levels of human ABR [26] while another found that resistance in hospitals could be better prevented by interventions simultaneously targeting transmission and antibacterial exposure [33] - however none of these studies considered ABU within a human-animal-environment One Health framework.

The current NSP-AMR in Thailand (2017–2021) aims to reduce ABU and transmission simultaneously, which according to our results is the most efficient way of reducing the burden of ABR in humans. We show that successfully achieving and maintaining current targets [25] until 2040 would result in a reduction of 6.0–18.8% in the number of people carrying resistant bacteria (assuming a 20% increase in sanitary practices to decrease transmission through ABR public knowledge). This impact is reasonable but could be improved by further reductions of human ABU (from 20% to 30%) (NSP-AMR; 8.5–24.9%). Alternatively, halving all transmission events alone (which depend on sanitary practices) without NSP-AMR targets being met was almost as effective in reducing ABR (5.7–25.6%). This shows that there are multiple alternatives which could strengthen the current NSP-AMR in Thailand. One such alternative is a combined approach: the current NSP-AMR with 50% reductions in transmission (compared to no change in sanitary practices) would be 3.1-times as effective in reducing human colonisation with resistant bacteria (median 23.1% reduction over 20 years). Fig. S2 may also be compared to the observed reductions in antibiotic use of 40% in animals and 9% in humans following the implementation of the O'Neill report in the United Kingdom from 2013 to 2017 [31]. When projecting the potential impact of reducing antibiotic use in animals by 40% and 9% in humans (approximated at 10% in our Fig. S2) until 2040, there would only be a 4.4% reduction in human ABR. Again, this would substantially increase if sanitary interventions to reduce transmission were also implemented alongside reductions in antibiotic use (up to 44.2% with 75% reduction in transmission). This shows that the general impact of restricting ABU can be greatly enhanced in the context of reduced transmission across various settings.

While our model is initially developed for the context of Thailand (the case study for model parameterisation), these results are potentially generalisable to any country or region with a high prevalence of resistant bacteria, including other Southeast Asian countries (22% prevalence of faecal colonisation with ESBL producing *Enterobacteriaceae*), African countries (22%), West Pacific countries (46%) or eastern Mediterranean countries (15%) [18]. However, it would be unreasonable to assume that each of these geographical regions share the exact parameters used in our Thailand study. For example, almost half of the Thai population are employed in the agricultural sector [35] and 30.1% of people use water from natural sources [36]; while some countries have similar demography, it will be important to collect parameter estimates specific for each country and region. However, ABR research in LMICs is characterised by data gaps as well as variability in data reliability, sharing and capacity [37]. In the absence of such data [37], this initial model may be used as a first step in understanding and evaluating other LMICs' ABR strategies. Ideally, future studies would obtain country-specific data on ABR (particularly for LMICs) or regional data from these countries. Then, a similar data-driven approach could be used to predict and forecast future ABR interventions with higher degrees of certainty in each specific region.

## 5 Conclusions

Our model makes some important predictions which have direct implications for human health in the context of ABR. Our conceptual model identified that human antibacterials are the primary driver in human ABR, but that there are many such interacting drivers which, if targeted by the correct interventions, could have large implications for the wider ABR problem. Interventions which focus on reducing ABU in humans can yield much greater impact when run in parallel to improved hygiene and sanitation interventions. Future work is needed to develop this model framework and to capture high-resolution data on transmission events between humans, animals and the environment, and to quantify the effects of ABU within animals and the environment on human health. This model has allowed estimation of the impact of the Thai NSP-AMR and has suggested where greater ambition in its targets could significantly increase its potential impact on ABR. We anticipate that the results of this modelling study will stimulate further discussion on One Health interventions within Thailand and across other LMICs.

## Ethics statement

No ethical approval was required for this mathematical modelling work.

## Data accessibility statement

All model code is open source and available for download on GitHub https://github.com/rdbooton/OHDARTmodel.

## Authors' contribution statement

Conception and design of the study: RDB, KMET. Acquisition of data: RDB, MBA, KMET. Mathematical modelling: RDB, AM, KKR, ACS, LV, MBA, KMET. Coding and simulations: RDB. Analysis and interpretation of results: RDB, AM, NA, HB, EF, HL, SM, EP, KKR, WS, JS, ACS, LS, VT, LV, OH-DART study group, MBS, KMET. Writing and drafting of the manuscript: RDB, AM, NA, HB, EF, HL, SM, EP, KKR, WS, JS, ACS, LS, VT, LV, OH-DART study group, MBS, KMET. Approval of the submitted manuscript: RDB, AM, NA, HB, EF, HL, SM, EP, KKR, WS, JS, ACS, LS, VT, LV, OH-DART study group, MBS, KMET.

## Declaration of Competing Interest

Declarations of interest: none

## References

[1] O'Neill J. (2016). Tackling drug-resistant infections globally: Final report and recommendations The Review on Antimicrobial Resistance Chaired by Jim O’Neill.

[2] WHO (2014). Antimicrobial resistance. Global report on surveillance. World Heal. Organ..

[3] WHO (2015). Global Action Plan on Antimicrobial Resistance.

[4] Hernando-Amado S., Coque T.M., Baquero F., Martínez J.L. (2019). Defining and combating antibiotic resistance from One Health and Global Health perspectives. Nat. Microbiol..

[5] Van Boeckel T.P., Glennon E.E., Chen D., Gilbert M., Robinson T.P., Grenfell B.T., Levin S.A., Bonhoeffer S., Laxminarayan R. (2017). Reducing antimicrobial use in food animals. Science (80-. ).

[6] Van Boeckel T.P., Pires J., Silvester R., Zhao C., Song J., Criscuolo N.G., Gilbert M., Bonhoeffer S., Laxminarayan R. (2019). Global trends in antimicrobial resistance in animals in low- and middle-income countries. Science (80-. ).

[7] Holmes A.H., Moore L.S.P., Sundsfjord A., Steinbakk M., Regmi S., Karkey A., Guerin P.J., Piddock L.J.V. (2016). Understanding the mechanisms and drivers of antimicrobial resistance. Lancet.

[8] Laxminarayan R., Matsoso P., Pant S., Brower C., Røttingen J.A., Klugman K., Davies S. (2015). Access to effective antimicrobials: a worldwide challenge. Lancet.

[9] World Health Organization (2012). The Evolving Threat of Antimicrobial Resistance: Options for Action.

[10] Woolhouse M., Ward M., Van Bunnik B., Farrar J. (2015). Antimicrobial resistance in humans, livestock and the wider environment. Philos. Trans. R. Soc. B Biol. Sci..

[11] Lipsitch M., Samore M.H. (2002). Antimicrobial use and antimicrobial resistance: a population perspective. Emerg. Infect. Dis..

[12] Wernli D., Jørgensen P.S., Morel C.M., Carroll S., Harbarth S., Levrat N., Pittet D. (2017). Mapping global policy discourse on antimicrobial resistance. BMJ Glob. Heal..

[13] Smith E. (2016). Evaluation of the Action Plan against the Rising Threats from Antimicrobial Resistance.

[14] White A., Hughes J.M. (2019). Critical importance of a One Health approach to antimicrobial resistance. Ecohealth.

[15] Huttner A., Harbarth S., Carlet J., Cosgrove S., Goossens H., Holmes A., Jarlier V., Voss A., Pittet D. (2013). Antimicrobial resistance: A global view from the 2013 World Healthcare-Associated Infections Forum. Antimicrob. Resist. Infect. Control.

[16] Murray C.J.L. (2012). Disability-adjusted life years (DALYs) for 291 diseases and injuries in 21 regions, 1990-2010: a systematic analysis for the Global Burden of Disease Study 2010. Lancet.

[17] Allegranzi B., Nejad S.B., Combescure C., Graafmans W., Attar H., Donaldson L., Pittet D. (2011). Burden of endemic health-care-associated infection in developing countries: systematic review and meta-analysis. Lancet.

[18] Karanika S., Karantanos T., Arvanitis M., Grigoras C., Mylonakis E. (2016). Fecal colonization with extended-spectrum beta-lactamase-producing Enterobacteriaceae and risk factors among healthy individuals: a systematic review and metaanalysis. Clin. Infect. Dis..

[19] Niewiadomska A.M., Jayabalasingham B., Seidman J.C., Willem L., Grenfell B., Spiro D., Viboud C. (2019). Population-level mathematical modeling of antimicrobial resistance: a systematic review. BMC Med..

[20] Sumpradit N. (2017). New chapter in tackling antimicrobial resistance in Thailand. BMJ.

[21] Phumart P., Phodha T., Thamlikitkul V., Riewpaiboon A., Prakongsai P., Limwattanon S. (2012). Health and economic impacts of antimicrobial resistance in Thailand. J. Health Serv. Res. Policy.

[22] Pongpech P., Naenna P., Taipobsakul Y., Tribuddharat C., Srifuengfung S. (2008). Prevalence of extended-spectrum beta-lactamase and class 1 integron integrase gene intl1 in Escherichia coli from Thai patients and healthy adults. *Southeast Asian J. Trop. Med.* Public Health.

[23] Luvsansharav U.O., Hirai I., Nakata A., Imura K., Yamauchi K., Niki M., Komalamisra C., Kusolsuk T., Yamamoto Y. (2012). Prevalence of and risk factors associated with faecal carriage of CTX-M β-lactamase-producing enterobacteriaceae in rural Thai communities. J. Antimicrob. Chemother..

[24] Boonyasiri A., Tangkoskul T., Seenama C., Saiyarin J., Tiengrim S., Thamlikitkul V. (2014). Prevalence of antibiotic resistant bacteria in healthy adults, foods, food animals, and the environment in selected areas in Thailand. Pathog. Glob. Health.

[25] Ministry of Public Health, Ministry of Agriculture and Cooperatives. 2016 National Strategic Plan on Antimicrobial Resistance 2017-2021, Thailand. (doi:10.2471/BLT.16.179648).

[26] van Bunnik B.A.D., Woolhouse M.E.J. (2017). Modelling the impact of curtailing antibiotic usage in food animals on antibiotic resistance in humans. R. Soc. Open Sci..

[27] Pal C., Bengtsson-Palme J., Kristiansson E., Larsson D.G.J. (2016). The structure and diversity of human, animal and environmental resistomes. Microbiome.

[28] Sasaki T. (2010). High prevalence of CTX-M β-lactamase-producing enterobacteriaceae in stool specimens obtained from healthy individuals in Thailand. J. Antimicrob. Chemother..

[29] Luvsansharav U.O. (2011). Analysis of risk factors for a high prevalence of extended-spectrum β-lactamase-producing Enterobacteriaceae in asymptomatic individuals in rural Thailand. J. Med. Microbiol..

[30] Thamlikitkul V., Tiengrim S., Thamthaweechok N., Buranapakdee P., Chiemchaisri W. (2019). Contamination by antibiotic-resistant bacteria in selected environments in Thailand. *Int. J. Environ. Res.* Public Health.

[31] HM Government (2019). Joint report on antibiotic use and antibiotic resistance, 2013–2017.

[32] Kristiansson E., Fick J., Janzon A., Grabic R., Rutgersson C., Weijdegård B., Söderström H., Joakim Larsson D.G. (2011). Pyrosequencing of antibiotic-contaminated river sediments reveals high levels of resistance and gene transfer elements. PLoS One.

[33] Knight G.M., Costelloe C., Deeny S.R., Moore L.S.P., Hopkins S., Johnson A.P., Robotham J.V., Holmes A.H. (2018). Quantifying where human acquisition of antibiotic resistance occurs: a mathematical modelling study. BMC Med..

[34] Tacconelli E. (2018). Discovery, research, and development of new antibiotics: the WHO priority list of antibiotic-resistant bacteria and tuberculosis. Lancet Infect. Dis..

[35] Coyne L. (2019). Characterizing antimicrobial use in the livestock sector in three south east asian countries (Indonesia, Thailand, and Vietnam). Antibiotics.

[36] Khamsarn S., Nampoonsak Y., Busamaro S., Tangkoskul T., Seenama C., Rattanaumpawan P., Boonyasiri A., Thamlikitkul V. (2016). Epidemiology of antibiotic use and antimicrobial resistance in selected communities in Thailand. J. Med. Assoc. Thail..

[37] Ashley E.A., Shetty N., Patel J., Van Doorn R., Limmathurotsakul D., Feasey N.A., Okeke I.N., Peacock S.J. (2019). Harnessing alternative sources of antimicrobial resistance data to support surveillance in low-resource settings. J. Antimicrob. Chemother..

