# Supplementary material for: One Health drivers of antibacterial resistance: Quantifying the relative impacts of human, animal and environmental use and transmission
Source: One Health. 2021 Jan 26;12:100220. doi: 10.1016/j.onehlt.2021.100220 (PMC7892992; doi:10.1016/j.onehlt.2021.100220)
Supplement: Supplementary material [file mmc1.docx]

**Supplementary Information**

**One Health drivers of antibacterial resistance: quantifying the relative impacts of human, animal and environmental use and transmission**

Ross D. Booton, Aronrag Meeyai, Nour Alhusein, Henry Buller, Edward Feil, Helen Lambert, Skorn Mongkolsuk, Emma Pitchforth, Kristen K. Reyher, Walasinee Sakcamduang, Jutamaad Satayavivad, Andrew C. Singer, Luechai Sringernyuang, Visanu Thamlikitkul, Lucy Vass, OH-DART study group, Matthew B. Avison, Katherine M.E. Turner

Katherine M.E. Turner

Email: [*Katy.Turner@bristol.ac.uk*](mailto:Katy.Turner@bristol.ac.uk)

**This file includes:**

Figure S1

Figure S2

Table S1

Table S2

*Sensitivity of assumptions relating to transmission*

Table S3

**
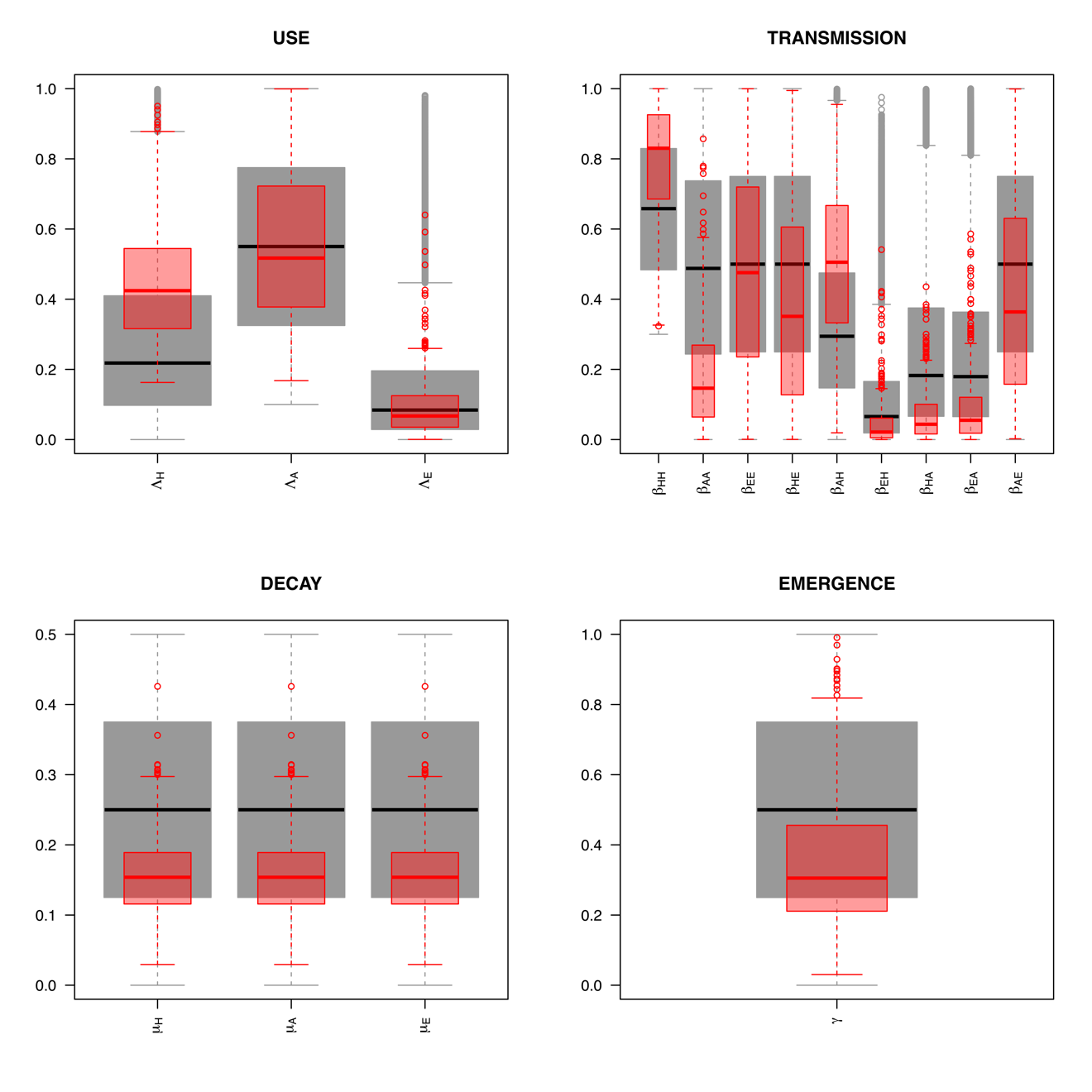
Fig. S1:** Box and whisker plot of parameter prior and posterior distributions. Grey boxes represent the search range/prior distribution of parameters (taken from Table 1), while red boxes represent the distribution of the best fits (from a Latin Hypercube sample LHS of 1 million), with interquartile range shaded, median, and minimum and maximum values. The best fits were obtained by applying the rules from Table S2 and parameter ranges to form prior distributions, from which the model was run 1 million times. The outputs from the model were then compared to the prevalance observed in humans, animals and the environment from Table 1 – rejecting those parameter sets which resulted in prevalence outside of those ranges. This resulted in a range of different fits which can replicate the prevalence data while capturing uncertainty in our parameter estimates.

**
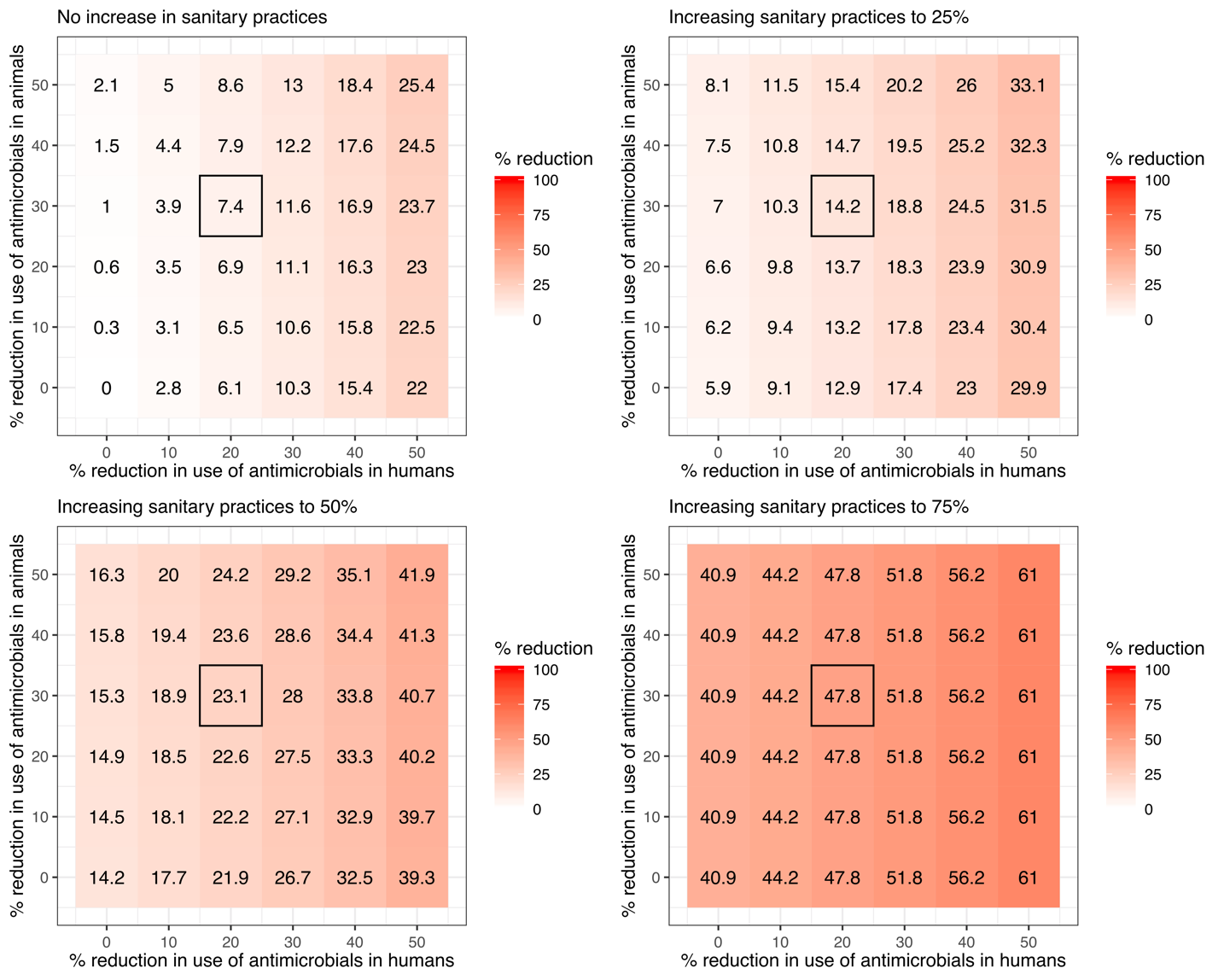
**

**Fig. S2:** The relationship between reducing transmission via increasing knowledge of sanitary measures and antibacterial use in humans and animals. Black square indicates the current National Strategic Plan on Antimicrobial Resistance (2017-2021) (NSP-AMR) targets for 0, 25, 50, 75 and 100% reductions in sanitary related transmission. With 20% reductions in use in humans, and 30% in animals, alongside no reduction in transmission related to education or prevention would reduce ABR in humans by 7.4% in 2040. A 25% increase in sanitary practices would result in 1.9-fold higher reductions in human ABR, 50% would result in 3.1-fold higher impact (23.1% reduction in human ABR) and 75% increases in sanitary practices would result in 6.5-fold higher impact (47.8% reduction in human resistance).

| ***Intervention*** | ***Parameters*** | ***Median reduction in colonisation with resistant bacteria in humans (95% credible interval) 2020-2040*** | ***Median reduction in colonisation with resistant bacteria in animals (95% credible interval) 2020-2040*** | ***Median reduction in colonisation with resistant bacteria in the environment (95% credible interval) 2020-2040*** |
| --- | --- | --- | --- | --- |
| No antibacterial use and no transmission | $\Lambda_{H}=\Lambda_{A}=\Lambda_{E}=0$  $\beta=0$ | 95.4 (65.7 – 99.7) | 95.4 (66.5 – 99.7) | 96.0 (77.9 – 99.7) |
| No antibacterial use | $\Lambda_{H}=\Lambda_{A}=\Lambda_{E}=0$ | 95.4 (65.7 – 99.7) | 95.4 (66.5 – 99.7) | 96.0 (77.9 – 99.7) |
| No human antibacterial use | $\Lambda_{H}=0$ | 95.4 (65.7 – 99.7) | 3.1 (0.1 – 24.4) | 32.9 (3.4 – 83.8) |
| No human antibacterial use and no human to human, human to environment, human to animal transmission | $\Lambda_{H}=0$  $\beta_{HH}=\beta_{HE}=\beta_{HA}= 0$ | 95.4 (65.7 – 99.7) | 3.5 (0.1 – 25.4) | 44.3 (5.4 – 92.2) |
| Increasing rate of loss of resistance in humans to max | $\mu_{H}= 1$ | 66.7 (51.2 – 79.5) | 2.3 (0.1 – 11.8) | 26.5 (3.4 – 54.8) |
| No transmission | $\beta=0$ | 38.5 (15.6 – 74.0) | 14.2 (0.6 – 61.6) | 96.0 (77.9 – 99.7) |
| No human to human, human to environment, human to animal transmission | $\beta_{HH}=\beta_{HE}=\beta_{HA}= 0$ | 17.9 (8.2 – 41.2) | 3.5 (0.1 – 25.4) | 44.3 (5.4 – 92.2) |
| No human to human transmission | $\beta_{HH}= 0$ | 17.1 (8.2 – 36.3) | 0.5 (0.0 – 6.4) | 6.2 (0.6 – 21.6) |
| No antibacterial use in animals and environment, no animal to animal, animal to human, animal to environment, no environment to animal, environment to human, environment to environment transmission | $\Lambda_{A}=\Lambda_{E}=0$  $\beta_{AA}=\beta_{AH}=\beta_{AE}= 0$  $\beta_{EA}=\beta_{EH}=\beta_{EE}= 0$ | 8.3 (1.3 – 17.7) | 95.4 (66.5 – 99.7) | 96.0 (77.9 – 99.7) |
| No antibacterial use in animals and environment, no animal to animal, animal to environment, no environment to animal, environment to environment transmission | $\Lambda_{A}=\Lambda_{E}=0$  $\beta_{AA}=\beta_{AE}= 0$  $\beta_{EA}=\beta_{EE}= 0$ | 7.2 (1.0 – 17.4) | 95.4 (66.5 – 99.7) | 96.0 (77.9 – 99.7) |
| No animal antibacterial use and no animal to human, animal to environment, animal to animal transmission | $\Lambda_{A}=0$  $\beta_{AH}=\beta_{AE}=\beta_{AA}= 0$ | 8.1 (1.3 – 17.2) | 95.4 (66.5 – 99.7) | 37.9 (7.8 – 87.9) |
| No animal to animal, animal to human, animal to environment transmission | $\beta_{AA}=\beta_{AH}=\beta_{AE}= 0$ | 8.1 (1.3 – 17.2) | 8.9 (0.3 – 33.5) | 37.9 (7.8 – 87.9) |
| No animal antibacterial use | $\Lambda_{A}=0$ | 7.1 (1.0 – 16.8) | 95.4 (66.5 – 99.7) | 29.1 (5.4 – 76.5) |
| No human to animal, animal to human transmission | $\beta_{HA}=\beta_{AH}= 0$ | 7.9 (1.1 - 17.1) | 3.1 (0.1 – 24.7) | 3.8 (0.4 – 16.2) |
| No animal to human transmission | $\beta_{AH}= 0$ | 7.9 (1.1 - 17.1) | 0.2 (0.0 – 2.5) | 2.6 (0.2 – 9.3) |
| Increasing rate of loss of resistance in animals to max | $\mu_{A}= 1$ | 5.3 (0.9 – 9.4) | 72.1 (59.8 – 82.3) | 25.1 (5.5 – 60.9) |
| No environment antibacterial use and no environment to human, environment to environment, environment to animal transmission | $\Lambda_{E}=0$  $\beta_{EH}=\beta_{EE}=\beta_{EA}= 0$ | 0.1 (0.0 – 1.8) | 0.9 (0.0 – 6.4) | 96.0 (77.9 – 99.7) |
| No environment to animal, environment to human, environment to environment transmission | $\beta_{EA}=\beta_{EH}=\beta_{EE}=0$ | 0.1 (0.0 – 1.8) | 0.9 (0.0 – 6.4) | 10.9 (0.8 – 47.8) |
| No environment to human, environment to animal, animal to environment, human to environment transmission | $\beta_{EH}=\beta_{EA}=\beta_{AE}$  $=\beta_{HE}= 0$ | 0.1 (0.0 – 1.8) | 0.9 (0.0 – 6.4) | 91.9 (49.7 – 99.3) |
| No environment to human, human to environment transmission | $\beta_{EH}=\beta_{HE}= 0$ | 0.1 (0.0 – 1.5) | 0.2 (0.0 – 3.7) | 41.9 (3.1 – 92.0) |
| No environment to human transmission | $\beta_{EH}= 0$ | 0.1 (0.0 – 1.4) | 0.0 (0.0 – 0.1) | 0.0 (0.0 – 0.5) |
| No environment antibacterial use | $\Lambda_{E}=0$ | 0.1 (0.0 – 1.8) | 0.8 (0.0 – 6.1) | 96.0 (77.9 – 99.7) |
| Increasing rate of loss of resistance in the environment to max | $\mu_{E}= 1$ | 0.1 (0.0 – 1.4) | 0.7 (0.0 – 5.0) | 83.7 (69.3 – 93.1) |
| No animal to animal transmission | $\beta_{AA}= 0$ | 0.4 (0.0 – 3.2) | 8.0 (0.3 – 30.7) | 2.2 (0.1 – 15.7) |
| No human to environment transmission | $\beta_{HE}= 0$ | 0.0 (0.0 – 0.8) | 0.2 (0.0 – 3.7) | 41.9 (3.1 – 92.0) |
| No environment to animal, animal to environment transmission | $\beta_{EA}=\beta_{AE}= 0$ | 0.1 (0.0 – 0.9) | 0.9 (0.0 – 6.3) | 34.4 (2.1 – 86.9) |
| No animal to environment transmission | $\beta_{AE}= 0$ | 0.0 (0.0 – 0.6) | 0.2 (0.0 – 2.7) | 34.4 (2.1 – 86.9) |
| No environment to environment transmission | $\beta_{EE}= 0$ | 0.0 (0.0 – 0.3) | 0.1 (0.0 – 1.4) | 10.6 (0.4 – 47.1) |
| No human to animal transmission | $\beta_{HA}= 0$ | 0.2 (0.0 – 2.2) | 3.1 (0.1 – 24.7) | 0.9 (0.0 – 11.5) |
| No environment to animal transmission | $\beta_{EA}= 0$ | 0.0 (0.0 – 0.5) | 0.9 (0.0 – 6.3) | 0.2 (0.0 – 2.2) |

**Table S1:** Reduction in colonisation with resistant bacteria in humans, animals and the environment from 2020-2040, impact on burden of resistant bacteria for combinations of use and transmission and loss of resistance.

| Assumption | Notation |
| --- | --- |
| Transmission from the environment to animals is greater than environment to humans due to higher proportions of shared bacterial genera in wastewater and animals (compared to wastewater and humans). | $\boldsymbol{\beta}_{\boldsymbol{EA}}\boldsymbol{>}\boldsymbol{\beta}_{\boldsymbol{EH}}$ |
| Transmission within populations of humans and animals is greater than the transmission between these populations and the transmission from the environment to these populations. | $\boldsymbol{\beta}_{\boldsymbol{HH}}\boldsymbol{>}\boldsymbol{\beta}_{\boldsymbol{AH}}\boldsymbol{,}\boldsymbol{\beta}_{\boldsymbol{EH}}$ |
|  | $\boldsymbol{\beta}_{\boldsymbol{AA}}\boldsymbol{>}\boldsymbol{\beta}_{\boldsymbol{HA}}\boldsymbol{,}\boldsymbol{\beta}_{\boldsymbol{EA}}$ |
| The majority of global ABU is within animals raised for food (73%), followed by human use. | $\boldsymbol{\Lambda}_{\boldsymbol{A}}\boldsymbol{>}\boldsymbol{\Lambda}_{\boldsymbol{H}}\boldsymbol{>}\boldsymbol{\Lambda}_{\boldsymbol{E}}$ |

Table S2: Summary of assumptions relating to transmission and use

**Sensitivity of assumptions relating to transmission**

In the main text, the transmission term e.g. ${\Lambda_{H}\beta}_{HH}H\left( 1-H \right)$ depends on the level of antibiotic present in the system (if $\Lambda=0$, then no transmission). A consequence of this assumption is that resistant bacteria do not transmit at all in the absence of antibiotics. Here, we explore an alternate model where transmission also occurs at a rate $\delta$, independent of the level of antibiotic. We model this assumption as a baseline transmission in the absence of antibiotic so that each term becomes $\left( \Lambda+\delta\right).$ This model can be expressed as:

$$\frac{dH}{dt}={\gamma\Lambda}_{H}\left( 1-H \right)+({\Lambda_{H}+\delta)\beta}_{HH}H\left( 1-H \right)+{{(\Lambda}_{H}+\delta)\beta}_{AH}A\left( 1-H \right)+{{(\Lambda}_{H}+\delta)\beta}_{EH}E\left( 1-H \right)-\mu_{H}H$$

$$\frac{dA}{dt}={\gamma\Lambda}_{A}\left( 1-A \right)+{{(\Lambda}_{A}+\delta)\beta}_{AA}A\left( 1-A \right)+{{(\Lambda}_{A}+\delta)\beta}_{HA}H\left( 1-A \right)+(\Lambda_{A}+\delta)\beta_{EA}E\left( 1-A \right)-\mu_{A}A$$

$$\frac{dE}{dt}={(\Lambda}_{E}+\delta)\beta_{EE}E\left( 1-E \right)+{(\Lambda}_{E}+\delta)\beta_{HE}H\left( 1-E \right)+{{(\Lambda}_{E}+\delta)\beta}_{AE}A\left( 1-E \right)-\mu_{E}E$$

We explore 2 additional scenarios with $\delta$= 0.01 and 0.1.

|  | *Median reduction in colonisation with resistant bacteria in humans (95% credible interval) 2020-2040* | | |
| --- | --- | --- | --- |
| *Intervention or scenario* | *Original model* $\boldsymbol{\delta=0}$ | $\boldsymbol{(\Lambda+\delta}$*) assumption with* $\boldsymbol{\delta=0.1}$ | $\boldsymbol{(\Lambda+\delta}$*) assumption with* $\boldsymbol{\delta=0.01}$ |
| NSP-AMR, Thailand | 12.2 (6.0 – 18.8) | 9.9 (4.5 – 15.4) | 11.9 (5.9 – 18.4) |
| NSP-AMR with 30% reduction in human ABU | 16.7 (8.5 – 24.9) | 12.7 (5.9 – 20.7) | 16.3 (8.3 – 24.4) |
| NSP-AMR with 30% increase in sanitary practices | 15.1 (7.4 – 24.6) | 13.1 (6.2 – 19.9) | 14.9 (7.3 – 24.1) |
| 50% reduction in all transmission only | 13.4 (5.7 – 25.6) | 12.9 (5.7 – 23.6) | 13.4 (5.7 – 25.4) |
| 50% reduction in water related transmission only | 0.1 (0.0 – 1.2) | 0.2 (0.0 – 1.5) | 0.1 (0.0 – 1.3) |
| 20% reduction in human ABU with 50% reduction in human-human transmission | 13.9 (7.9 – 22.3) | 11.4 (6.0 – 19.5) | 13.6 (7.8 – 22.0) |
| 20% reduction in human ABU with 95% reduction in transmission from sewage/manure | 6.3 (3.5 – 8.6) | 4.6 (2.2 – 7.2) | 6.1 (3.3 – 8.5) |

Table S3: The median reduction in human ABR for various scenarios given changes in the transmission assumption. The smaller the value of $\boldsymbol{\delta}$, the closer the results are to the original model, which is expected (as original model has implicit $\boldsymbol{\delta}$ = 0). Even if baseline transmission parameter in the absence of antibiotic was an additional 10%, then the impact is fairly similar to the original set of results.
